# Elastomeric Fire and Heat-Protective Materials Containing Functionally Active Microheterogeneous Systems

**DOI:** 10.3390/polym16152163

**Published:** 2024-07-30

**Authors:** Vladimir G. Kochetkov, Daria A. Kryukova, Daniil A. Urzhumov, Oksana M. Novopoltseva, Natalia A. Keibal, Vladimir Burmistrov, Victor F. Kablov

**Affiliations:** 1 Department of Chemical Technology of Polymers and Industrial Ecology, Volzhsky Polytechnic Institute (Branch) of Volgograd State Technical University, 42a Engelsa St., Volzhsky 404121, Russia; 2 Department of Organic Chemistry, Volgograd State Technical University, 28 Lenina Avenue, Volgograd 400005, Russia; crus_himself@mail.ru

**Keywords:** aluminosilicate microfibers, aluminosilicate microspheres, fire-retardant properties, heat resistance, physical–mechanical properties, sizing, phosphorus–boron–nitrogen-containing modifier

## Abstract

This research aims to explore how functionally active structures affect the physical, mechanical, thermal, and fire-resistant properties of elastomeric compositions using ethylene–propylene–diene rubber as a base. The inclusion of aluminosilicate microspheres, microfibers, and a phosphorus–boron–nitrogen–organic modifier in these structures creates a synergistic effect, enhancing the material’s heat-insulating properties by strengthening coke and carbonization processes. This results in a 12–19% increase in heating time for unheated sample surfaces and a 6–17% increase in residual coke compared to existing analogs. Microspheres help counteract the negative impact of microfibers on composition density and thermal conductivity, while the phosphorus–boron–containing modifier allows for controlling the formation of the coke layer.

## 1. Introduction

The development of heat-resistant materials using elastomers that can endure high-temperature conditions for short durations is a crucial objective for safeguarding structures across diverse industries, including aviation, rocketry, and the oil-and-gas sector [1,2]. These materials find application in coating rocket engine combustion chambers and nozzles, as well as in gas generator casings and other related equipment.

Researchers Bhuvaneswari, Walter, and Ahmed have been conducting investigations into the development of fire-resistant polymer materials [3,4,5]. Fire-heat composite materials (FHPMs) are subject to a wide array of demanding criteria, often presenting conflicting requirements: improving one parameter may lead to the degradation of other properties. Scientists are confronted with a complex, multifaceted challenge in finding an optimal balance that enables the creation of the most efficient material. The effectiveness of fire-retardant materials hinges on the physicochemical transformations of components, their thermal decomposition, and alterations in the material’s chemical structure [6,7]. Processes involving structuring and the formation of a protective layer with low thermal conductivity are activated under the influence of high temperatures [8,9,10,11].

In most compositions designed for ablative protection against high temperatures, inorganic additives play a pivotal role by aiding in the formation of carbon coke during material thermal decomposition. To enhance coke formation processes, components containing elements such as halogens, phosphorus, nitrogen, boron, metals, or combinations thereof are introduced [12,13,14,15].

Recently, researchers have increasingly used mixtures of different flame retardants or substances in which molecules simultaneously contain elements such as phosphorus, boron, and nitrogen to achieve a higher effectiveness in reducing the flammability of materials.

The mechanism of action of the coating containing the mentioned compounds is based on the fact that, when exposed to flames, as well as during the polymer material’s destruction and oxidation, phosphorus–boron-containing compounds form polyphosphoric and boron-containing acids. These acids distribute as a film on the material’s surface and hinder the ingress of oxygen necessary to sustain the combustion process. Moreover, a porous glassy coating of polyphosphoric acid, which has low thermal conductivity, develops on the surface of the polymer, thereby decreasing the level of heat that enters the polymer’s interior. Phosphorus–boron-containing compounds promote reactions of cyclization, condensation, and carbonization of the decomposition products during combustion, leading to the formation of a “coke cap”. During the pyrolysis of polymers containing phosphorus compounds, phosphoric acid and its anhydrides are formed, which catalyze dehydration, dehydrogenation, and contribute to carbonization.

Phosphorus–organic compounds primarily act in the condensed phase, altering the direction of the decomposition processes and increasing the coke residue, while reducing the amount of gaseous combustible products [6]. Some types of phosphorus-containing flame retardants decompose to form gaseous compounds, wherein phosphorus facilitates the formation of sooty conglomerates, reducing combustion completeness. The introduction of phosphorus-containing fragments into polymer compositions not only reduces their flammability but often enhances adhesion, corrosion resistance, and other beneficial properties.

However, the range of highly effective phosphorus-based flame retardants produced by the global industry is insufficient and can be expanded through the synthesis of new synergistic phosphorus–nitrogen–boron–halogen-containing compounds.

Furthermore, the application of fire-protective coatings on composite and elastomeric materials, considering aspects such as adhesion, thermal stability, and the effects of static and dynamic mechanical loads, remains incompletely studied.

In challenging operational environments, fire- and heat-resistant materials face not only high temperatures and pressures but also rapid gas flow, resulting in the erosion of the surface layer of ablative thermal protection materials (FHPM), material thinning, and a subsequent decrease in effectiveness. Addressing this issue involves incorporating microfibrous fillers such as kaolin, basalt, carbon fibers, and similar materials to create a reinforcing framework, enhancing the material’s ability to withstand erosion. The introduction of these fillers into polymers leads to diverse interactions at the polymer–filler interface, impacting the composite material’s mechanical properties, physicochemical stability, and thermal resistance [16,17,18].

However, the use of these fillers can result in technological problems, such as fiber agglomeration, reduced material homogeneity, and increased density and thermal conductivity. To address these issues, various treatments can be applied to improve the uniform distribution of microfibers and enhance interaction with the polymer. The phosphorus–boron–nitrogen-containing modifier (PBN) has been shown to be an effective system for improving physical and mechanical properties, as well as fire-retardant and thermophysical characteristics of the composite.

In References [19,20], chemical processes in flames upon the introduction of phosphorus-containing additives; transformation mechanisms of additives; and mechanisms of the influence of these compounds on the combustion rate, structure, limits of spread of hydrogen, and hydrocarbon flames are considered. Flame inhibition by phosphorus-containing additives was explained by the authors of [20,21] by an increase in the rate of recombination of hydrogen proton and hydroxide anion and reactions with phosphorus oxides and oxyacids. The described processes are characteristic of the gas phase.

The action in the condensed phase consists of the fact that, upon the decomposition of the flame retardant, residues of phosphoric acid are formed that act as a dehydrating agent, promoting the formation of carbonized structures. At the same time, aerosols may also form, contributing to the deactivation of radicals through a wall effect. It has been noted in works [22,23] that, in this phase, phosphorus compounds and their decomposition products cause dehydration of the polymeric structure, cyclization, crosslinking, aromatization, and graphitization; in other words, they act as crosslinking agents. Polyphosphoric acids are formed on the surface of the coating.

As mentioned in [24], these compounds can act in both phases. For example, when phosphorus-containing flame retardants are added to polystyrene, the rate of its decomposition in the condensed phase increases (rather than decreases, as one might expect), and triphenylphosphine and sulfur exhibit a synergistic effect in inhibiting the combustion of polystyrene.

It should be noted that microencapsulated phosphorus can be used as a modifying agent, and when introduced into epoxy polymers, an increase in their fire resistance is observed, while the physical–mechanical and dielectric properties remain practically unchanged [17].

The application of a product based on melamine, aminotrimethylenephosphonic acid, and FeCl_3_·6H_2_O (2d-CFA) as an effective modifier for polymer compositions is well-known [25]. The “labyrinth” effect of 2d-CFA nanosheets is beneficial for hindering mass and heat transfer during the early pyrolysis of PLA.

In Reference [26], a novel bio-based intumescent flame retardant (PP-Fe) with a nanosheet structure was successfully fabricated using simple self-assembly technology. The resulting material exhibited high mechanical properties and flame retardancy.

Modified nanotubes based on aluminosilicates have been applied [27] to enhance the fire safety and thermostability of polylactic acid polymers.

## 2. Results

Depending on the ratio of microspheres and microfibers, we expected the formation of different types of functional–active structures. The investigated ratios of microspheres, microfibers, and FAS are presented in Table 1.

**Figure 1:**
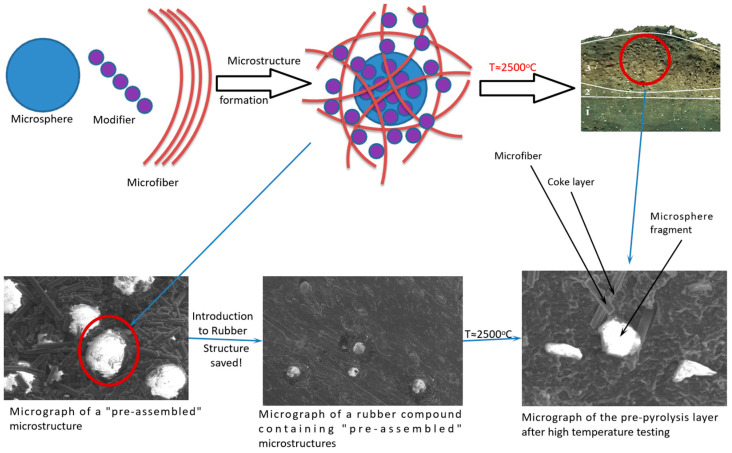
Scheme of “preliminary assembly” of functional–active structures and their microphotographs.

Using electron scanning microscopy, the formation of structures (Figure 1) was established, with a microsphere at the center surrounded by a microfiber. The presence of phosphorus atom peaks in the elementograms confirms that the surface of microdispersed components was altered through modification.

**Figure 2:**
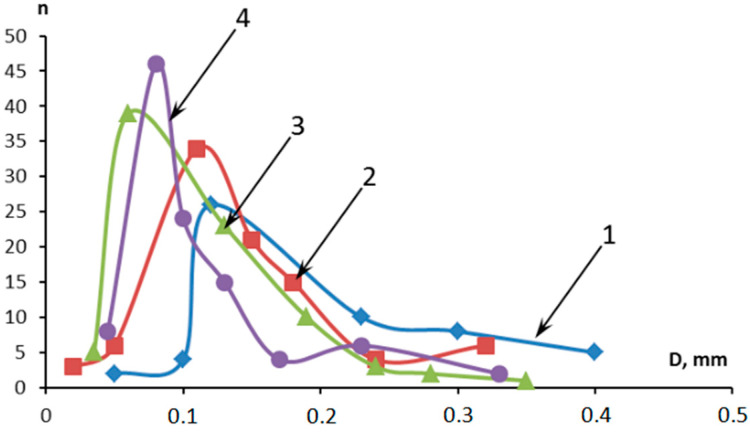
Distribution of pores in the pre-pyrolysis layer: 1—control sample; 2—5MKV:10MSF; 3—10MKV:5MSF; and 4—15MKV:5MSF.

The microphotograph (Figure 2) shows that the functional–active structures (FASs) introduced retain their original form. Additionally, the treated microspheres or microfibers, which exist as intermediate variations, contribute positively to the fire-resistant properties of the material.

Table 2 shows the characteristics of cured samples of the studied rubber compositions.

## 3. Discussion

The introduced functional–active components, forming relatively large microstructures, lead to a slight decrease in strength indicators, but their values still remain above the normative values (Table 2). More importantly, as the content of microspheres increases, the density of the compositions decreases and tends to align with that of the control sample. The reduction of density is significant in creating FHPMs for aviation and rocket technology [28].

Samples that contain a ratio of 5 microspheres to 10 microfibers with functional–active structures demonstrate the most effective fire-resistant characteristics.

As heat moves through the fire-resistant material, various adaptive processes take place: the upper layers of the material experience degradation of the polymer matrix, while the added functional–active components aid in creating a denser porous coke layer strengthened with microfibers (see Figure 2). The addition of phosphorus–boron–nitrogen–organic modifiers on the surface of microspheres in the deeper layers of the material triggers the formation of coke, which leads to a slower heating rate.

If we assume that the coke residue is formed solely from the mineral part of the recipe, the introduction of 5–10 wt.

To evaluate the performance retention of fire-resistant composite materials containing a combination of modified aluminosilicate microspheres and microfibers at elevated temperatures (75–150 °C), elastic and tensile properties were determined using a Shimadzu AG-X Plus tensile testing machine equipped with a thermal chamber.

As shown in Table 3, the introduction of a combination of microfibers and microspheres in a ratio of 10:5 provides the highest retention of material properties.

The manufacturing technology of fire-resistant materials involves a multilayer structure, and strong bonding between the layers is achieved by using an appropriate adhesion promoter. The presence of nitrogen in the synthesized compound suggests that it may exhibit adhesive activity and interact with segments of the film-forming polymer macromolecules.

**Figure 3:**
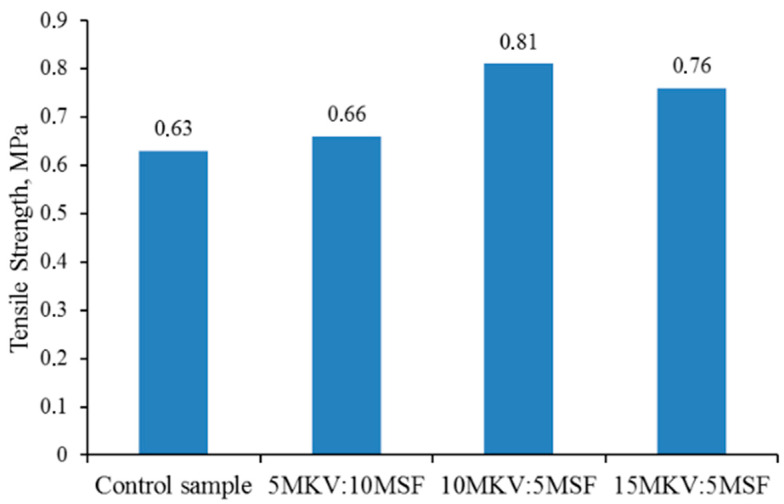
Bonding strength of rubbers based on EPDM-40 containing special polychloroprene glue grade 88SA.

The results of comparative analysis show that the introduction of a modifying additive increases the adhesive strength. The highest adhesive strength is achieved when bonding rubber compounds based on ethylene–propylene rubber with adhesive 88SA, with the addition of 5–10 wt. (Figure 3).

Further increasing the content of the modifier does not significantly affect the strength characteristics, which may be associated with a weakening of the diffusive nature of the interaction between the adhesive and the substrate.

The synthesized modifier in elastomeric material can also act as a co-agent of adhesion, contributing to the strengthening of bond strength in the adhesive–elastomer system. Its presence improves diffusive processes during bonding.

**Figure 4:**
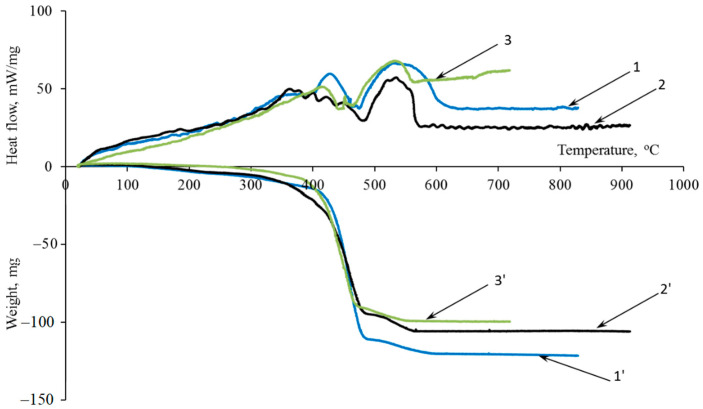
(1–3) DTA and (1′–3′) TG analysis curves: (1) control sample, (2) 10MKV:5MSF sample, and (3) 15MKV:5MSF sample.

The effectiveness of the investigated additives is confirmed by differential thermal analysis (DTA) and thermogravimetric (TGA) analyses (Figure 4).

The amount of energy expended on structuring processes, material coking, modifier decomposition, and its chemical changes due to heat can be assessed by examining the area beneath the endothermic peak on the DTA curve. The introduction of functional–active structures leads to an increase in coke residue by 4–30% and an increase in the area under the endothermic peak by 24%.

**Figure 5:**
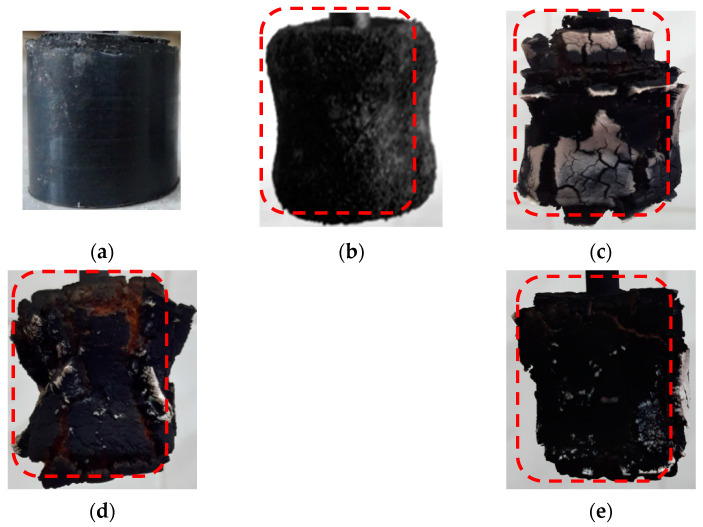
The high-speed heat flow effects on the surface of the sample: (**a**) control sample before testing, (**b**) control sample after testing, (**c**) 5MKV:10MSF, (**d**) 10MKV:5MSF, and (**e**) 15MKV:5MSF. The red dotted line shows the initial size of the samples.

In Figure 5, the tested samples after erosion-resistance testing under high-speed heat flow conditions are presented. The control sample (Figure 5b) is characterized by a significant mass loss and a short ignition time. The presence of functional–active structures intensifies coke-formation processes, while microfiber contributes to the creation of a strong coke with low thermal conductivity (Figure 5e).

**Figure 6:**
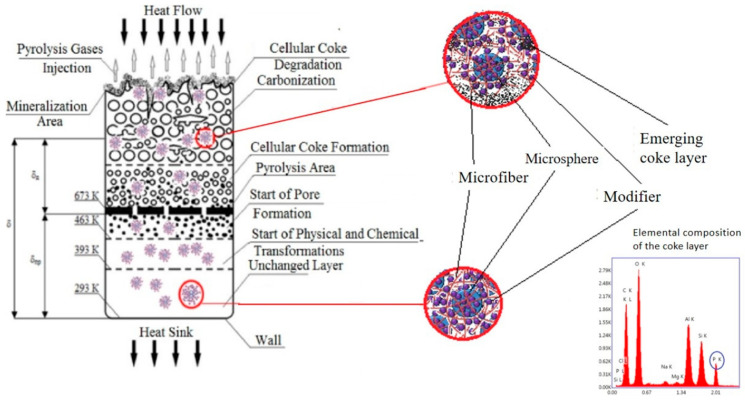
Proposed mechanism of fire-protection action of materials containing functional–active structures.

Based on the experimental findings, we were able to formulate a hypothesis regarding the mechanism through which materials containing functional–active structures provide fire protection (Figure 6).

When exposed to a high-temperature flow, the following zones can be distinguished within the cross-section of an FRCM [29]:

1. Zone of intense thermal destruction of the coke layer in direct contact with the gas flow (temperature ranging from 2500 to 4000 °C): In this zone, thermal destruction of foamed coke occurs, leading to its mineralization, volatilization of inorganic compounds, and carbon itself (above 3700 °C). The gas pyrolysis zone is also affected by erosion caused by the gas flow.

2. Zone of coking and foamed coke formation: In this zone, the polymer undergoing pyrolysis undergoes coking, resulting in the formation of a porous coke structure. The size of the pores in the plastic zone increases, starting from the polymer binder pyrolysis zone at temperatures above 300 °C, or for ethylene–propylene rubbers, above 400 °C. When the coke loses its plasticity at temperatures above 500–600 °C, some micro-cracking and delamination of the foamed coke occur (stratification in microzones). Gas pyrolysis products flow out through the formed pores into the high-temperature zone.

3. Zone of pyrolysis of the polymer binder at temperatures ranging from 300 to 400 °C: In this zone, an approximately 1 mm thick thermal decomposition of the polymer (pyrolysis) takes place. Chemical bonds in high-molecular-weight macromolecules of rubber and other polymers and resins introduced into the FHPM are broken, resulting in the formation of low-molecular-weight products (pyrolysis gases) and a sharp loss of material mass. As viscosity sharply decreases in this zone, pore formation and the development of a porous structure begin. Comparatively small pores are formed here, and they increase in size with temperature until the material retains certain plastic properties (pore size exceeding 0.3 mm). The thickness of the sample sharply increases during pore formation. Deformation processes occurring in the samples intensify pore formation, alter pore shape, and create through-channels.

The formation of coke occurs in already-foamed rubber, and its porous structure is largely determined by the process of pore formation in rubber. Additionally, fillers, plasticizers, and modifiers influence the coking process by catalyzing carbonization (acting as centers for crystallization, leading to dehydrogenation reactions, and modifying the ratio of volatile pyrolysis fractions).

4. Pre-pyrolysis layer: Processes of thermodecomposition of weak chemical bonds begin at temperatures above 523 K, and at temperatures around 673 K, pore-formation processes start in partially destructured materials. Simultaneously, materials based on butadiene–nitrile rubbers and other diene polymers undergo structuring and cyclization processes, resulting in material shrinkage (523–573 K), while materials based on chlorinated polyethylene transition to a thermoplastic state within this temperature interval.

During this temperature interval, the growth of pores begins, originating from nucleation bubbles on filler particles and microdroplets of plasticizer. Nucleation pores have sizes smaller than 1 μm. The growth of bubbles occurs due to the diffusion of gaseous decomposition products of rubber and dissolved or adsorbed water within them.

5. Zone of material heating from the pore formation zone to the wall. During the initial stages of heating (up to 373–423 K), thermal expansion of the material occurs, along with melting processes of certain crystalline components and the formation of nucleation pores under the influence of volatile components.

Depending on the heating conditions, the nature of the rubber, and the coating thickness, local thermal explosions can occur in FHPM, leading to irregular destruction of the porous rubber layer and coke. The impact of the gas flow, thermal deformations in the carbonizing FHPM consisting of layers with different moduli, leads to the cracking of the coke layer and the formation of through-cracks and -channels through which pyrolysis gases escape (the “blowout effect”).

The coking process strongly depends on the type of material; in structuring materials, a dense network of the polymer matrix is formed, promoting the creation of dense fine–porous coke. In destructuring materials made of thermoplastic, coarse–porous rubber, loose, weak coke is formed that is easily destroyed by the gas flow.

At the same time, the heat-protection function is not only related to coke formation but also to the thermal costs of rubber thermodegradation. In this regard, materials based on EPDM have a significant advantage over materials based on butadiene–nitrile rubbers, where thermal transformations are accompanied by significant effects of structuring and cyclization. The protective effect is also associated with a number of other physicochemical transformations in the sample, such as melting, sublimation, boiling of components, chemical reactions of modifiers, significant influence on processes of pore formation, thermodegradation, pyrolysis, and coking of fillers, plasticizers, vulcanizing agents, and other components of FHPM.

Under the influence of high-temperature heat flow, the introduced functional–active components transform into a reinforced coke layer with increased resistance to erosion and reduced thermal conductivity. In this case, microspheres act as coke-formation centers. The process is initiated by the PBN modifier layer on the microspheres’ surface, which captures and holds the microfibers essential for enhancing the strength of coke when subjected to material erosion conditions.

Under high-temperature exposure in the coking zone, melting and destruction of microspheres occur, but even on the surface of fragments, microfibers are retained and continue to perform their function of reinforcing coke.

## 4. Materials and Methods

The materials under investigation in this study were rubbers based on triple ethylene–propylene–diene rubber (EPDM-40 produced by Nizhnekamsk Synthetic Rubber Plant). This choice was made due to its relatively low density, degree of unsaturation, and high heat resistance [30]. Aluminosilicate microspheres were obtained from the INOTECK Group of Companies (Moscow, Russia). The microsphere size is 0–50 µm, with a wall thickness of 10% of the diameter. Aluminosilicate microfibers were obtained from the Closed Joint-Stock Company “Scientific and Production Enterprise Izomat” (Solnechnogorsk, Russia). The average diameter of the microfibers is 2.10 µm.

The compositions being studied are outlined in Table 4. Previous research [11,14] has identified the optimal weight percentages of microspheres, microfibers, and an organic element modifier at 5, 10, and 3 wt pts., respectively, per 100 wt pts of rubber.

As demonstrated in [11], the incorporation of hollow aluminosilicate microspheres in elastomeric fire-retardant materials enhances the efficiency of these compositions by reducing thermal conductivity and density, while maintaining optimal physicomechanical properties. The proposed mechanism suggests that additional crosslinked structures are formed within the rubber matrix due to the interaction between the polymer matrix and the microspheres. An increase in the microsphere content leads to improved filler–filler interactions. The constant value of the elastic component of the shear modulus at high deformations indicates the constancy of the contribution to the modulus of the hydrodynamic effect, polymer–filler interaction, and “structures within the rubber”.

The addition of fibers to elastomeric fire-retardant materials improves their efficiency under high-temperature conditions by creating a stronger protective coke layer on the surface. This contributes to an increase in the time required to heat the unheated sample surface to 100 °C and a reduction in mass loss and linear burning rate [14].

For comparison purposes, control samples of elastomeric compositions without functionally active additives were prepared. The properties of compositions containing individual components of functionally active structures were previously investigated [11,14].

The rubber compounds were prepared in two stages. In the first stage, a high-speed laboratory micro-mixer of the “Brabender” type (Polimermash, Saint Petersburg, Russia) was used to compound the masterbatch at a temperature of 105–110 °C, where a calculated amount of the functionally active modifier was added and homogenized. In the second stage, sulfur, vulcanization accelerators, and activators were added on laboratory rollers (Polimermash, Saint Petersburg, Russia) at a temperature of 45–50 °C after a 24-h rest. Vulcanization of the samples was then performed in a PHG-2 212/4 vulcanizing press, (Carver, Aldridge, Hungary) under the determined optimum mode, using a flow meter MDR 3000 Professional (MonTech, Columbia City, IN, USA) (165 °C, 40 min).

The physical and mechanical properties of standard elastomer samples were determined using a tearing machine Shimazu AG-Xplus 1.0 kN (Shimadzu, Kyoto, Japan), in accordance with ISO 37-2017 [31]. The cohesive strength of the compositions was determined in accordance with ISO 9026:2007, “Raw rubber or unvulcanized compounds—Determination of green strength” [32]. The influence of the functional–active systems’ content on the adhesive properties of the composition was evaluated according to ASTM D3163-01, “Standard Test Method for Determining Strength of Adhesively Bonded Rigid Plastic Lap-Shear Joints in Shear by Tension Loading”, using a standard adhesive composition based on polychloroprene glue grade 88SA (manufacturer: Novbytkhim JSC, Gatchina, Russia).

To assess the fire and heat resistance of the samples, the following parameters were determined according to the developed methodology: the dependence of the temperature on the unheated surface of the sample on the exposure time of an open-flame plasma torch, sample mass loss, and linear burning rate. The sample used was a disc with a diameter of 100 mm and a thickness of 10 mm. A temperature of about 2000 °C was created on the surface of the sample during high-temperature heating.

To evaluate the erosion resistance of the material under conditions of high-temperature exposure, the sample was placed on a rotating shaft and tangentially heated by a plasma torch. During the test, the ignition time of the sample and the time of coke delamination were recorded. The diameter of the sample after the test was then determined. The destructed layer was removed, and the thickness of the undestructed layer was determined. The strength characteristics of coke are determined taking into account the forces on detachment realized at the boundary between the coke layer and the undecomposed material at the moment of coke layer detachment under the action of centrifugal forces when rotating a cylindrical sample at a constant speed while being heated at a high temperature in the plasma torch flame. The strength of the coked layer upon detachment was determined by the Formula (1):

(1) $$\sigma =\frac{2\cdot {\left(\pi \cdot \omega \right)}^{2}\cdot \left(R_{0}^{2}-R^{2}\right)\cdot \rho_{Κ}}{{\left(R^{0}-R\right)}^{2}}$$

where ω is the angular velocity, rev/s; R_0_ is the initial radius, mm; R is the radius to the boundary of the pyrolysis layer where detachment occurs, mm; and p_c_ is the density of coke, kg/m^3^.

**Figure 7:**
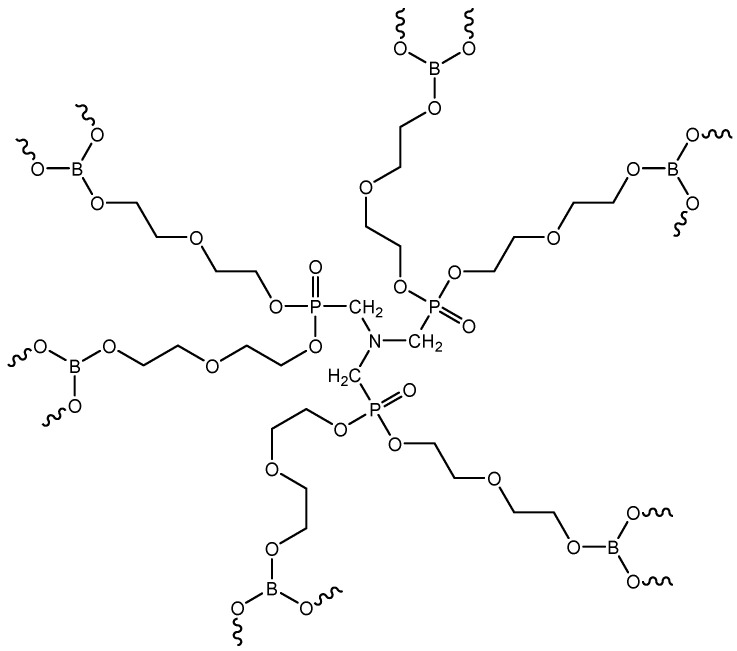
Structural formula of the synthesized PBN modifier.

The preliminary assembly of functional–active structures involved treating the surfaces of microspheres and microfibers with condensed compounds of nitrogen, phosphorus, and boron (the structural formula is shown in Figure 7). The synthesis and isolation of the PBN modifier were carried out according to the procedure described in Reference [33]. The main characteristics of the obtained product are presented in Table 5.

The scanning electron microscope Versa 3D (FEI Company, Hillsboro, OR, USA) was used to conduct morphological studies of the functionally active structures (FAS) and vulcanizates, as well as coke structures. This microscope allows for a detailed examination and analysis of the samples at a high resolution.

Differential thermal analysis (DTA) and thermogravimetric analysis (TGA) of the samples were performed using a derivatograph Q-1500D (MOM Szerviz Kft, Budapest, Hungary).

## 5. Conclusions

The findings suggest that incorporating pre-assembled functional–active systems into elastomeric fire-resistant materials improves their interaction with the elastomeric matrix, leading to more effective distribution. This targeted delivery of the modifier to the interfacial layer enhances coking processes, specifically at the interface. By introducing both microspheres and microfibers during coking initiation, structures are formed where microfibers encircle microspheres, strengthening the coke layer and enhancing its erosion resistance.

## Figures and Tables

**Table 1:** Proportions studied of microspheres, microfibers, and PBN.

Ingredient	Sample Number		
	5MKV:10MSF	10MKV:5MSF	15MKV:5MSF
	Content, wt. pts. Per 100 wt. pts. Rubber		
Aluminosilicate microspheres	10	5	5
Aluminosilicate microfibers	5	10	15
PBN modifier	3	3	3

**Table 2:** Characteristics of the developed elastomeric materials.

Parameter	Sample Number				
	Ref.	Control Sample	5MKV:10MSF	10MKV:5MSF	15MKV:5MSF
Tensile strength, *f_t_*, MPa	Not less than 6.0	15.9	10.5	12.7	14.54
Breaking elongation, ε_rel_, %	Not less than 300	445	420	370	400
Permanent Elongation, θ_perm_, %	Not more than 30	21	23	17	16
Density, ρ, kg m^−3^	Not more than 1100	1078	1061	1072	1095
Heating time of unheated surface of a sample up to 100 °C, s	–	62	72	89	92
Coke number CCV, %	–	12.3	16.7	17.8	18.6
Linear burning speed, *V_l.b._*, mm min^−1^	–	31.9	31.1	27.1	24.9
Coke layer-tear propagation Strength σ, mPa	–	36.8	39.2	42.5	42.5

**Table 3:** Investigation of strength characteristics of the composite material at elevated temperatures.

Sample Number	Tensile Strength, MPa			Breaking Elongation, %		
	75 °C	125 °C	150 °C	75 °C	125 °C	150 °C
Control sample	3.61	2.66	2.18	306	207	135
5MKV:10MSF	3.40	3.07	3.68	257	149	158
10MKV:5MSF	4.87	3.52	3.10	316	180	127
15MKV:5MSF	3.07	2.79	2.45	283	171	195

**Table 4:** Proportions of the studied elastomeric materials.

Ingredient	Sample Number			
	Control Sample	5MKV:10MSF	10MKV:5MSF	15MKV:5MSF
	Content, wt. pts. Per 100 wt. pts. Rubber			
EPDM-40	100	100	100	100
Precipitated silica BS-120	30	30	30	30
Zinc oxide	5	5	5	5
Stearin	1	1	1	1
Accelerator MBT	2	2	2	2
Sulfur	2	2	2	2
FAS	0	18	18	18
Total	140	156	156	156

**Table 5:** Physicochemical indicators of the synthesized PBN modifier.

Indicator	Value
Refractive index	1.4620
Viscosity coefficient	760 mm^2^/s
Appearance	Viscous oily yellow-orange liquid
Solubility in water	Soluble
Solubility in acetone	Insoluble

## Data Availability

The data presented in this study are available in the present article.
